# Salt forms of amides: protonation of acetanilide

**DOI:** 10.1107/S2053229624007332

**Published:** 2024-08-06

**Authors:** Harry S. Jaconelli, Alan R. Kennedy

**Affiliations:** a Department of Pure & Applied Chemistry, University of Strathclyde, 295 Cathedral Street, Glasgow G1 1XL, Scotland, United Kingdom; University of the Witwatersrand, South Africa

**Keywords:** crystal structure, acetanilide, pharmaceutical, salt selection, protonation, protonated amide

## Abstract

Treating the amide acetanilide (*N*-phenyl­acetamide, C_8_H_9_NO) with aqueous strong acids allowed the structures of five hemi-protonated salt forms of acetanilide to be elucidated. *N*-(1-Hy­droxy­ethyl­idene)anilinium chloride–*N*-phenyl­acetamide (1/1), [(C_8_H_9_NO)_2_H][Cl], and the bromide, [(C_8_H_9_NO)_2_H][Br], triiodide, [(C_8_H_9_NO)_2_H][I_3_], tetra­fluoro­borate, [(C_8_H_9_NO)_2_H][BF_4_], and di­iodo­bromide hemi(diiodine), [(C_8_H_9_NO)_2_H][I_2_Br]·0.5I_2_, analogues all feature centrosymmetric dimeric units linked by O—H⋯O hy­dro­gen bonds that extend into one-dimensional hy­dro­gen-bonded chains through N—H⋯*X* inter­actions, where *X* is the halide atom of the anion. Protonation occurs at the amide O atom and results in systematic lengthening of the C=O bond and a corresponding shortening of the C—N bond. The size of these geometric changes is similar to those found for hemi-protonated paracetamol structures, but less than those in fully protonated paracetamol structures. The bond angles of the amide fragments are also found to change on protonation, but these angular changes are also influenced by conformation, namely, whether the amide group is coplanar with the phenyl ring or twisted out of plane.

## Abstract 2

Treating acetanilide with aqueous strong acids gives protonation at the amide O atom and a series of anhydrous hemi-protonated salt forms of this simple amide.

## Introduction

The formation of salt phases of Active Pharmaceutical Ingredients (APIs) is a simple technique used by the pharmaceutical industry to enhance desirable materials properties of an acidic or basic API, whilst retaining the essential functional groups of the organic com­pound. Often the targeted property is enhanced aqueous solubility (Stahl & Wermuth, 2008 ▸). Salt screening is unlikely to be performed if the API contains only traditionally neutral functional groups, such as amides. All chemists learn at an early stage that amides are much less basic than amines, and it is perhaps this seed that leads to the misidentification or misnaming of amides as ‘non-ionizable’ (Manallack, 2007 ▸; Bethune *et al.*, 2009 ▸). Just because a com­pound is not normally ionized, or not protonated under normal physiological conditions, does not of course mean that it cannot be protonated given the right conditions. Protonation of amides is common in acidic solution and protonated forms of amides may even be isolated in the solid state to allow characterization by diffraction. In 2012, Nanubolu *et al.* (2012 ▸) surveyed known crystal structures of protonated amides. Since then, much API relevant work on the crystal structures of protonated amides has concentrated on car­bam­az­e­pine and its relatives (Perumalla & Sun, 2012 ▸, 2013 ▸; Buist *et al.*, 2013 ▸, 2015 ▸; Buist & Kennedy, 2016 ▸; Eberlin *et al.*, 2013 ▸) and, most importantly to the current work, on paracetamol (Perumalla & Sun, 2012 ▸; Perumalla *et al.*, 2012 ▸; Trzybiński *et al.*, 2016 ▸; Kennedy *et al.*, 2018 ▸; Suzuki *et al.*, 2020 ▸). Protonated amide cations are of course themselves strong acids and thus highly unlikely drug candidates. The inter­est in their isolation and characterization is thus driven by academic inter­est and by manufacturing considerations, such as utilizing their mechanical properties to change com­paction and tab­leting properties prior to final formulation (Perumalla *et al.*, 2012 ▸). Herein we report five crystal structures of hemi-protonated salt forms of the paracetamol congener acetanilide (ACT). Despite its historical use as a pharmaceutical and its structural similarity to paracetamol, ACT is no longer used as an API (Brodie & Axelrod, 1948 ▸). However, as a fundamental simple amide, it has multiple industrial uses, including as a synthetic precursor in the pharmaceutical industry (Singh *et al.*, 2019 ▸; Li *et al.*, 2024 ▸). The structures presented herein are [(ACT)_2_H][Cl], [(ACT)_2_H][Br], [(ACT)_2_H][I_3_], [(ACT)_2_H][BF_4_] and [(ACT)_2_H][I_2_Br]·0.5I_2_. Previous solid-state phase work on such a fundamental com­pound as ACT has been surprisingly limited. Structures previously reported include that of the com­pound itself (Wasserman *et al.*, 1985 ▸), those of a small number of cocrystal forms (*e.g.* Megumi *et al.*, 2013 ▸; Oliveira *et al.*, 2013 ▸), and a few structures where ACT acts as a ligand (*e.g.* Buchner & Müller,, 2023 ▸; Marchetti *et al.*, 2008 ▸).
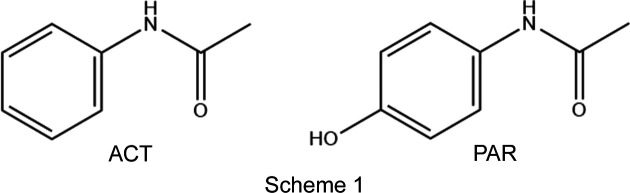


## Experimental

### Synthesis and crystallization

The I_3_, BF_4_ and mixed Br/I samples were obtained by dis­solving ACT (0.21 g, 1.6 mmol) in methanol (1 ml). Approximately 1 ml of the appropriate concentrated aqueous acid was then added. For the mixed Br/I species, this acid was a 5:1 mixture of HI and HBr. In each case, after 3–7 d of slow evaporation, crystals were deposited. In the case of the mixed Br/I species, orange crystals of the ACT salt form were mixed with colourless crystals of the decom­position product [PhNH_3_][Br]. Crystals of the Cl salt were prepared in a similar way, but here ACT (0.40 g, 3 mmol) was dissolved in methanol (4 ml) and concentrated HCl (2 ml) added. Using similar techniques with concentrated HBr gave only crystals of [PhNH_3_][Br]. Crystals of the required Br salt form were thus prepared by dissolving ACT (0.25 g) in concentrated aqueous HBr (2 ml). Crystals of the Br salt were deposited within 3 d.

### Refinement

Crystal data, data collection and structure refinement details are summarized in Table 1 ▸. Data measured for [(ACT)_2_H][Br] were treated as twinned by a 180° rotation about [0, 0.71, −0.71] to give a HKLF 5 formatted reflection file. The BASF parameter refined to 0.3996 (13). The structure of the mixed I/Br species was originally refined as [(ACT)_2_H][I_2_Br]·0.5I_2_, with all halide sites ordered. However, residual electron density and displacement parameters suggested that this treatment was not idealized. Individual trial calculations treating each halide site as a mixture of I and Br suggested that a mixed model was appropriate for two of the halide sites. Thus, the I5/Br3 and Br1/I7 sites were modelled as mixed occupancy, each with a total halide occupancy of one. See Section 3 (*Results and discussion*) for further comment. All H atoms bound to C atoms were placed in idealized positions and refined in riding modes, with C—H = 0.95 and 0.98 Å for CH and CH_3_ groups, respectively. *U*_iso_ values were set at 1.2*U*_eq_ or 1.5*U*_eq_ of the parent atom for CH and CH_3_ groups, respectively. H atoms bound to N or to O atoms were refined isotropically, but with the *X*—H distances restrained to 0.88 (1) Å. The exceptions to this latter statement were [(ACT)_2_H][Cl], where H atoms bound to N atoms were refined freely and isotropically, and [(ACT)_2_H][I_2_Br]·0.5I_2_, where the half-occupancy H atoms of the OH groups were placed in idealized positions.

## Results and discussion

**Figure 1:**
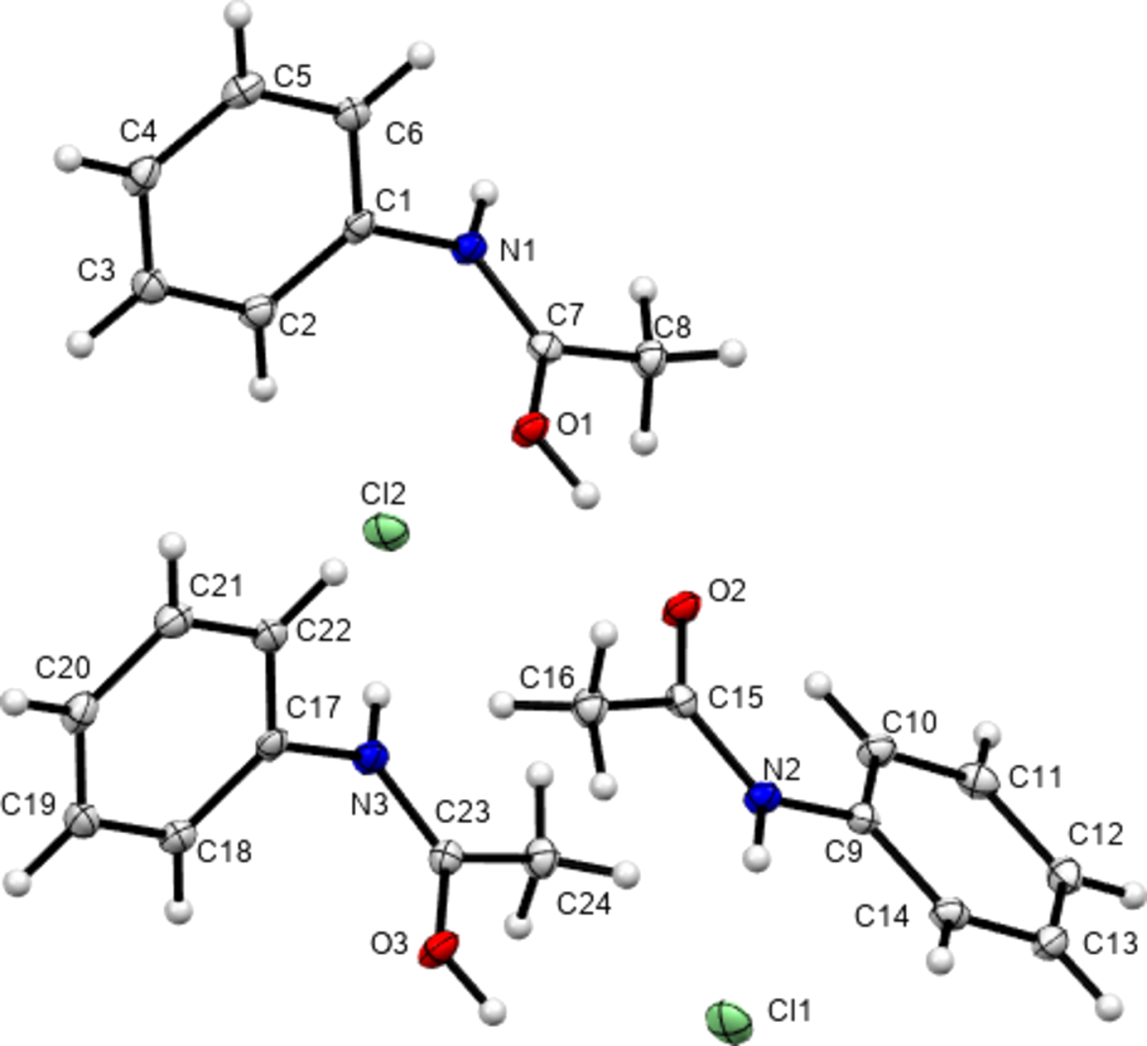
Contents of the asymmetric unit of [(ACT)_2_H][Cl]. Note that atom Cl1 sits on a crystallographic centre of symmetry. Here and elsewhere, displacement ellipsoids are drawn at the 50% probability level and H atoms are drawn as small spheres of arbitrary size.

In 2012, Perumalla & Sun (2012 ▸) reported that non-aqueous sources of HCl were capable of protonating amides that concentrated aqueous hydro­chloric acid could not, and that non-aqueous sources of HCl could give anhydrous versions of salt forms of amides where aqueous acid could not. It has been shown since that salt forms of amides (including anhydrous salts) are accessible by either simply dissolving the amide in the concentrated aqueous acid or by adding concentrated aqueous acid to alcohol solutions of the amide (Buist *et al.*, 2015 ▸; Kennedy *et al.*, 2018 ▸). The latter aqueous methods were adopted here using the strong acids H*X* (*X* = Cl, Br and I) and HBF_4_. All isolated crystals containing ACT were found to be anhydrous hemi-protonated salt forms. The asymmetric unit contents of the five structures [(ACT)_2_H][Cl], [(ACT)_2_H][Br], [(ACT)_2_H][I_3_], [(ACT)_2_H][BF_4_] and [(ACT)_2_H][I_2_Br]·0.5I_2_ are presented in Figs. 1 ▸–5 ▸ ▸ ▸ ▸, with selected crystallographic and refinement parameters given in Table 1 ▸. Note that for the mixed I/Br species, the formula used herein, [(ACT)_2_H][I_2_Br]·0.5I_2_, is a simplified approximation. Two of the halide atom sites were modelled as mixed I/Br sites, see Section 2.2 (*Refinement*). A small excess of I in the refined model implies the existence of some I_3_^−^ anions, as well as the majority I_2_Br^−^, and gives an overall formula of [(ACT)_2_H][I_2_Br]_0.93_[I_3_]_0.07_·0.5I_2_. All the halide atoms of this structure lie on the mirror plane of the space group *P*2_1_/*m*. Well-ordered organic structures containing mixed-halide anions such as I_2_Br are known (Buist & Kennedy, 2014 ▸), but, as here, structures with mixed I/Br polyhalides are often found to be disordered (*e.g.* Kobra *et al.*, 2019 ▸; Laukhina *et al.*, 1997 ▸). A recent article describes how the structures of polyiodide and related salt forms can be treated as being formed from *X*^−^, *X*_3_^−^ and *X*_2_ building blocks (Blake *et al.*, 2024 ▸).

All five structures are protonated at the amide O atom and all form O—H⋯O hy­dro­gen-bonded dimers between what have been modelled as protonated ACT(H) cations and neutral ACT mol­ecules. Note that, in all cases, free refinement of the H-atom position resulted in the H atom moving towards the centre of the O⋯O separation and that in the given models the acidic H atom has been restrained to sit 0.88 (1) Å from the O atom to which it was found to be closest. The very short O⋯O distances of 2.422 (10)–2.4650 (12) Å indicate strong inter­actions and it should be borne in mind that the models with distinct ACT(H) and ACT units may in fact represent cases where the H atom is inter­mediate between the two O-atom positions. A relevant precedent for such an inter­mediate behaviour is given by Eberlin *et al.* (2013 ▸). An obvious difference between paracetamol (PAR) and ACT is that, under a wide variety of aqueous, non-aqueous and even solid-grinding preparation methods, PAR tends to give fully protonated [PAR(H)][*X*] species, where *X* is Cl, Br, I or a variety of *R*SO_3_ anions (Perumalla *et al.*, 2012 ▸; Suzuki *et al.*, 2020 ▸; Trzybiński *et al.*, 2016 ▸; Kennedy *et al.*, 2018 ▸). In contrast, the only structures of PAR hemi-protonated forms reported are with Cl^−^ and I_3_^−^ anions (Perumalla & Sun, 2012 ▸; Kennedy *et al.*, 2018 ▸). That ACT was only found to form hemi-protonated salts may be due to a lower basicity of the amide caused by it lacking the electron-donating *para*-OH group of PAR. This conjecture is supported by the only other structure available with a protonated (Ar)NHC(O)Me fragment. Reaction of *o*-tolylNHC(O)Me, which has no strong electron-donating substituent, with nitric acid was found to give a hemi-protonated salt form (Gubin *et al.*, 1989 ▸). All hemi-protonated forms of PAR and of *o*-tolylNHC(O)Me feature the same dimer motif linked by O—H⋯O hy­dro­gen bonding between protonated and neutral amide functions, as is seen in the five ACT structures.

**Figure 6:**
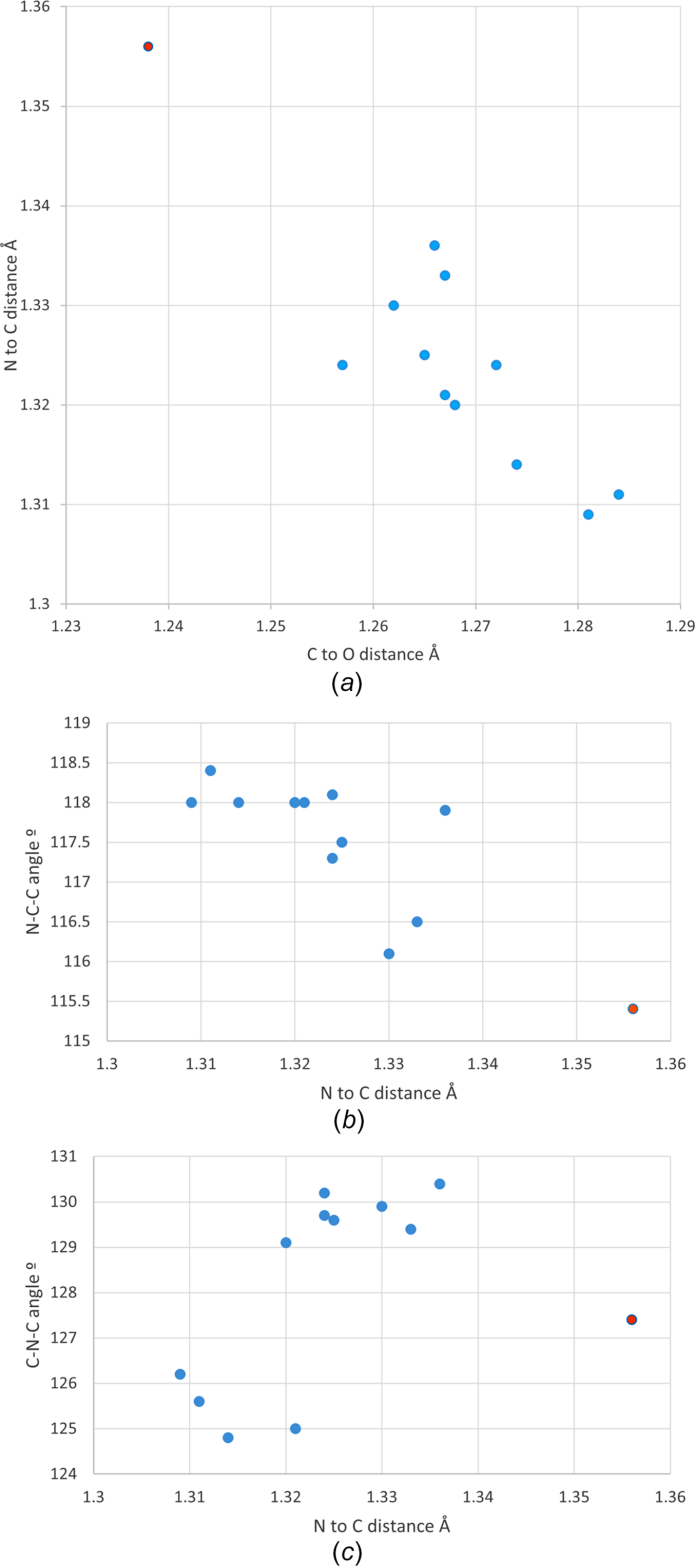
(*a*) Amide C—O and N—C distances in hemi-protonated ACT(H) structures. Amide N—C distances plotted against (*b*) N—C—C and (*c*) C—N—C angles. Blue dots = hemi-protonated ACT species from this article and orange dots = neutral ACT from the high-resolution charge–density data set of Hathwar *et al.* (2011 ▸).

**Figure 7:**
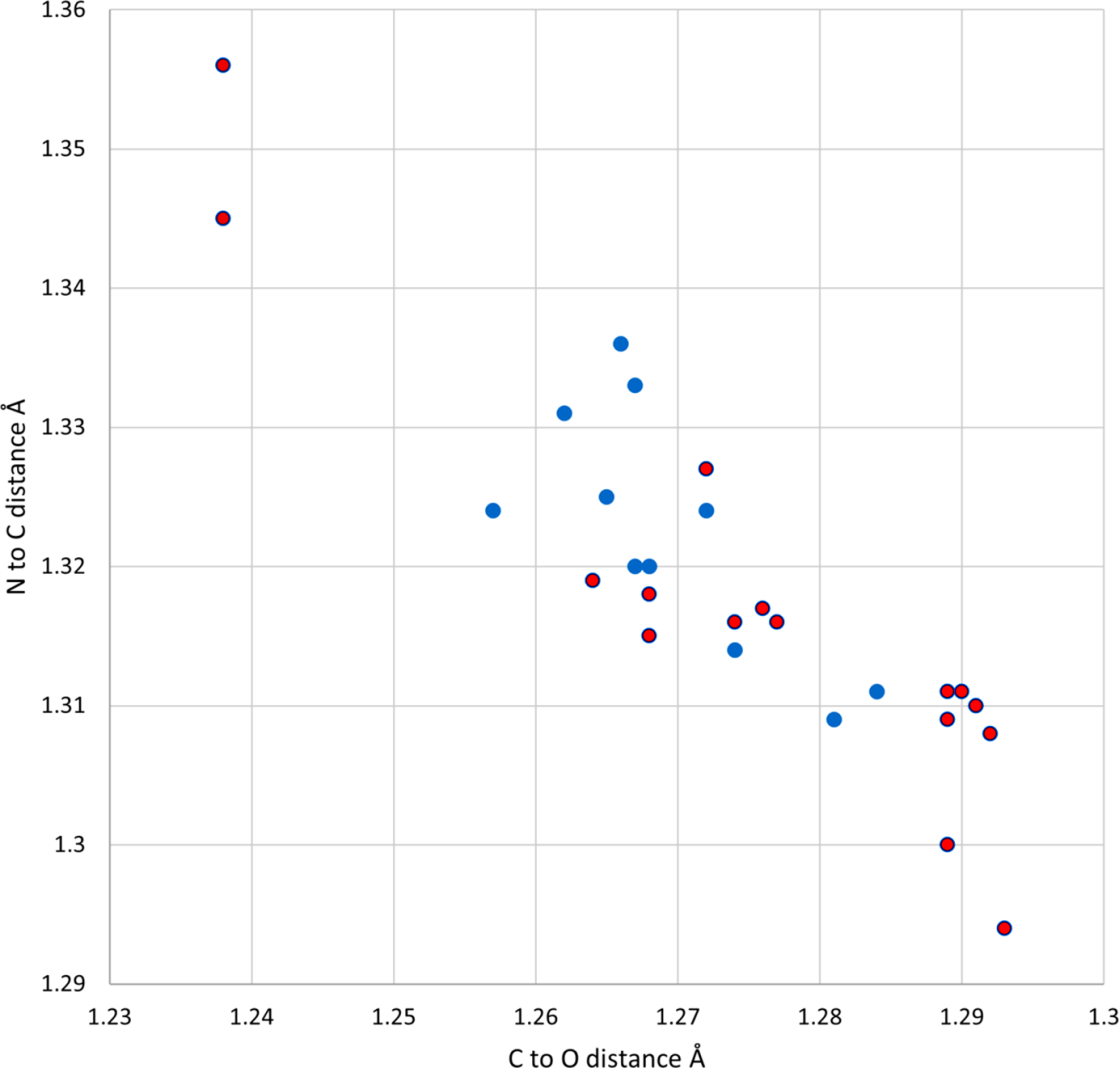
Amide C—O and N—C distances in ACT and PAR structures. Blue dots = hemi-protonated ACT species from this article, orange dots = neutral ACT and PAR (Nichols & Frampton, 1998 ▸), and red dots = hemi- and fully-protonated PAR species.

Protonation of the amide in car­bam­az­e­pine has been shown to lengthen the amide C—O bond and shorten the amide C—N bond, as would be expected for a structure showing resonance of the N-atom lone pair through to the O atom (Buist *et al.*, 2013 ▸; Eberlin *et al.*, 2013 ▸). Similar geometric changes can be seen in the amide groups of the five protonated ACT structures (see Fig. 6 ▸). The C=O bond distances lengthen from 1.238 Å in ACT to a range of 1.257 (5)–1.2843 (15) Å for the ACT(H) species. This is accom­panied by a corresponding decrease in the C—N distance from 1.356 Å in ACT to 1.3114 (16)–1.336 (10) Å for ACT(H). That all crystallographically independent ACT fragments show changes in bond lengths indicate that all are affected to some degree by protonation – despite the structural models formally containing both ACT and ACT(H) fragments. Comparison of Fig. 6 ▸ with Fig. 7 ▸ shows that these changes are com­parable with those found in the structures of hemi-protonated PAR salt forms (Perumalla & Sun, 2012 ▸; Kennedy *et al.*, 2018 ▸). They are also similar to the geometric changes caused by coordination of ACT to main group or transition metals (Megumi *et al.*, 2013 ▸; Oliveira *et al.*, 2013 ▸). Fig. 7 ▸ also shows a cluster of points below and to the right of the hemi-protonated species, these are the fully-protonated PAR salt forms which can be seen to show larger deviations from the geometries of the parent APIs than do the hemi-protonated salt forms (Perumalla *et al.*, 2012 ▸; Suzuki *et al.*, 2020 ▸; Trzybiński *et al.*, 2016 ▸; Kennedy *et al.*, 2018 ▸).

The bond angles of the amide group can also be seen to change upon protonation. The N—C—CH_3_ angle widens from 115.4° in ACT to between 116.1 (1) and 118.4 (1)° in the ACT(H) fragments. Correspondingly, the O—C—N angle decreases from 123.4° in ACT to between 120.1 (1) and 121.9 (1)° for ACT(H). Plotting N—C—CH_3_ against the N—C bond length (Fig. 6 ▸) shows that the values for two of the ACT(H) fragments are displaced somewhat towards the values for ACT. Inter­estingly, these displaced values are for the single crystallographically independent ACT moiety in the Cl and Br structures that are modelled as being nonprotonated. Thus, despite undoubtably having an inter­mediate-type geometry, these moieties modelled as ACT are truly more ACT-like than are those modelled as ACT(H). The same is not true of the fragment modelled as ACT in the BF_4_ structure, and careful examination of the residual electron density suggests that the ACT/ACT(H) model adopted here is indeed less suitable and that both fragments should perhaps bear a partial H atom.

A final inter­esting feature shown in Fig. 6 ▸ is that, on protonation of the amide, the C—N—C angles do not either uniformly widen or narrow. Instead, both behaviours are observed [C—N—C for ACT of 127.4° *versus* the range 124.8 (3)–130.4 (6)° for the ACT(H) salt forms]. This causes the ACT(H) structures to split into two separate clusters when, for instance, the C—N—C angle is plotted against the N—C distance. The four points where the angle has narrowed correspond to the four fragments with nonplanar conformations, whilst the larger group all have the amide group approximately coplanar with the aromatic ring (com­pare twist angles of 49.1–57.5 ° for the nonplanar group to twist angles of 2.0–13.6° for the planar group). A similar effect can be seen in the protonated PAR structures from the literature. Of 14 crystallographically independent PAR units, ten are planar and these all show an increase in the C—N—C angle upon protonation. Four structures of protonated PAR structures, those with sulfonate anions, adopt twisted conformations and for these the C—N—C angle remains constant or decreases upon protonation. Presumably an out-of-plane twist reduces steric contacts between the amide fragments and the rings, allowing the angles about the N atom to narrow.

**Figure 8:**
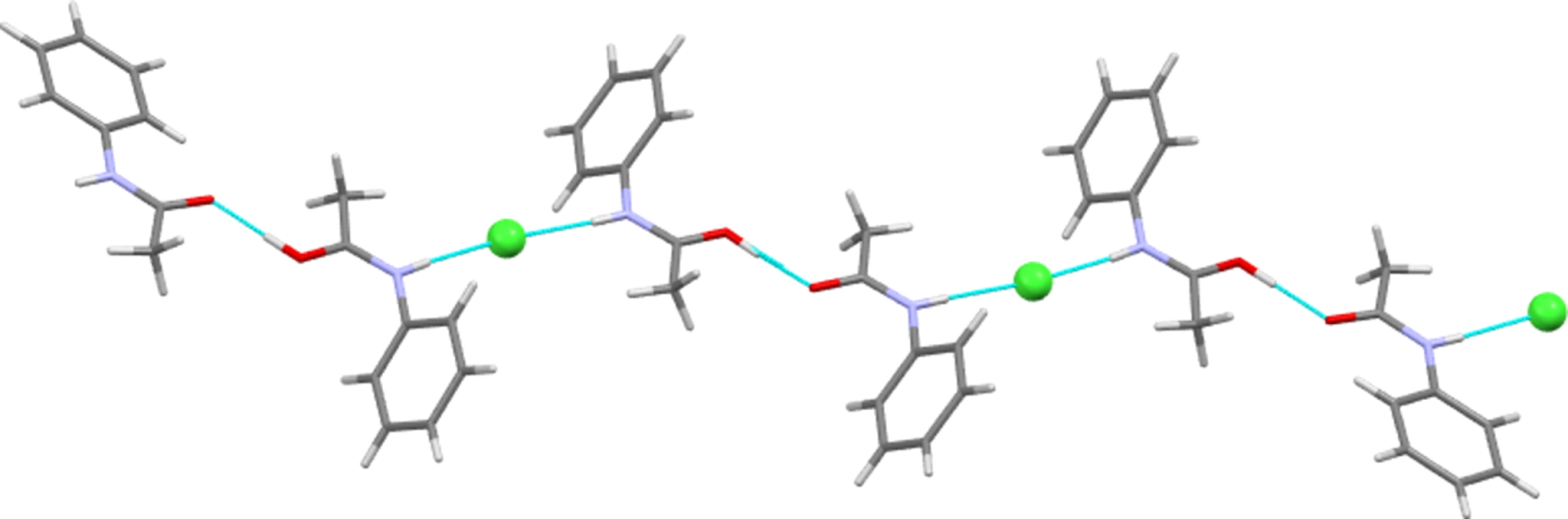
The one-dimensional hy­dro­gen-bonded chain formed by O—H⋯O and N—H⋯Cl contacts in [(ACT)_2_H][Cl]. Similar chains are present in the Br and I/Br salt structures.

**Figure 9:**
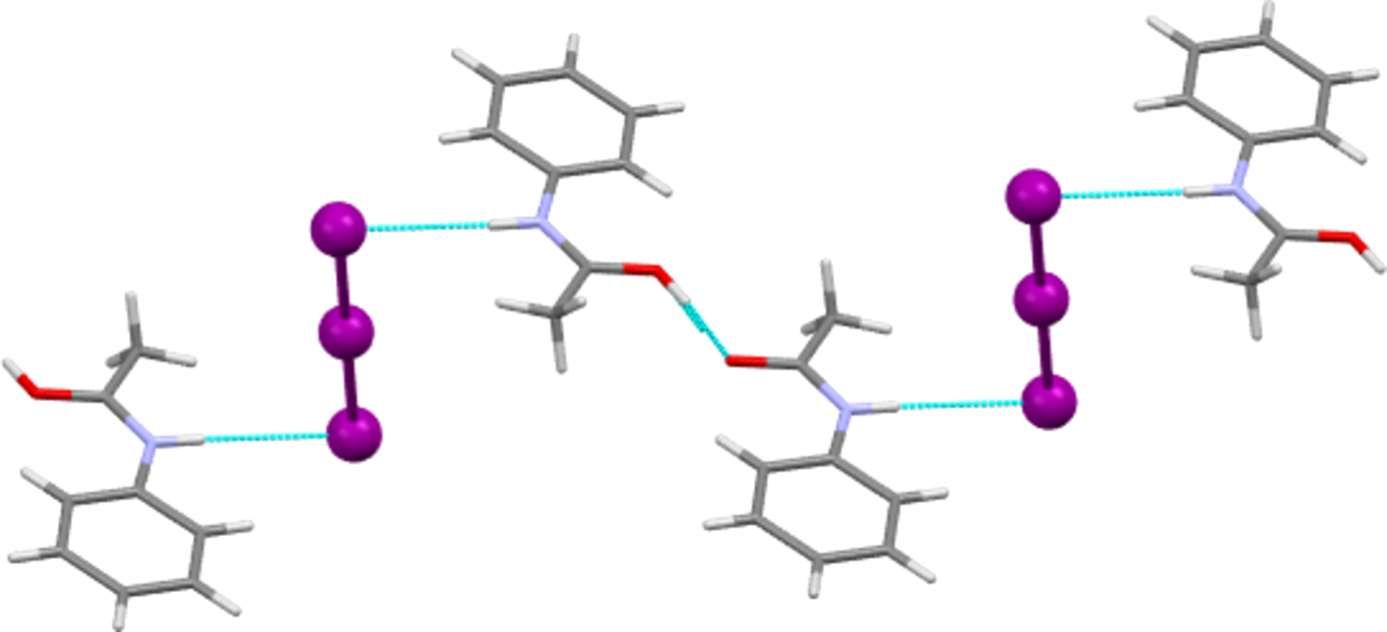
The stepped one-dimensional hy­dro­gen-bonded chain formed by O—H⋯O and N—H⋯I contacts in [(ACT)_2_H][I_3_]. A similar chain motif is present in [(ACT)_2_H][BF_4_], but here the steps caused by the linear I_3_^−^ anion are absent.

As described above, the main inter­molecular feature of each hemi-protonated ACT structure is the strong centrosymmetric O—H⋯O hy­dro­gen-bond contact that forms dimers between ACT mol­ecules and ACT(H) cations. This dimer is also seen in the structures of other hemi-protonated amides. Another inter­molecular contact common to all five ACT species herein is that the N—H group always acts as a hy­dro­gen-bond donor to the anion (see Tables 2 ▸–6 ▸ ▸ ▸ ▸ for details). In the Cl, Br and mixed I/Br salt forms, the N—H to anion inter­actions link the dimers described above to give 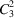
(10) one-dimensional hy­dro­gen-bonded chains (see Fig. 8 ▸ for an example). In the Cl and Br salts, each chain involves all crystallographically unique mol­ecules and ions, whilst in the mixed I/Br structure, it is only the Br site of each I_2_Br^−^ anion that accepts hy­dro­gen bonds from N—H and each crystallographically unique ACT fragment propagates a separate hy­dro­gen-bonded chain. The I_3_ and BF_4_ salt forms display equivalent chain motifs, but, in these cases, a three-atom link through the body of the anion replaces the single halide atom link above (see Fig. 9 ▸). Thus, these are formally 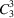
(12) motifs.

**Figure 10:**
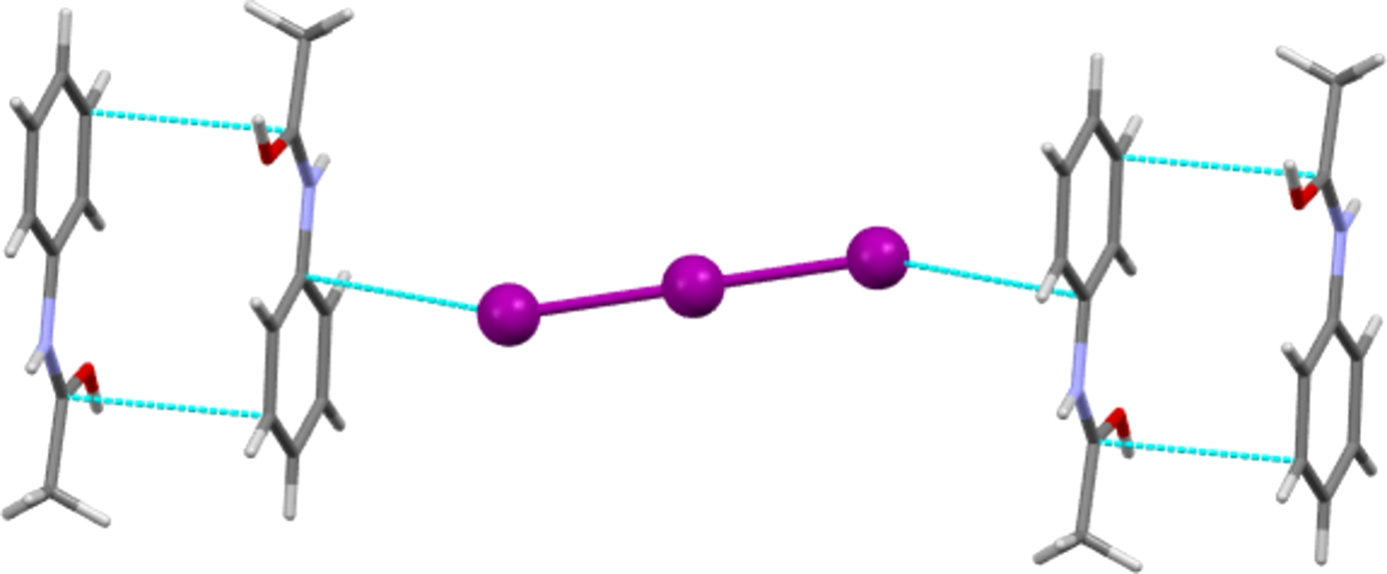
Section of the structure of [(ACT)_2_H][I_3_], showing both anti­parallel π–π contacts between ACT fragments and π-geometry C⋯I contacts between the amide and the anion.

Other inter­molecular contacts of around the sum of the van der Waals radii or less are more variable. The I_3_, BF_4_ and mixed I/Br salt forms all feature π–π inter­actions between ACT fragments [minimum C⋯C distances of 3.330 (7), 3.355 (2) and 3.348 (11) Å for the I_3_, BF_4_ and mixed I/Br salts, respectively]. For the latter species, this is between offset parallel ACT units, and for the I_3_ and BF_4_ salt forms, this is between anti­parallel units. Fig. 10 ▸ shows the π–π inter­actions of [(ACT)_2_H][I_3_] and also illustrates the last type of inter­molecular contact to be highlighted here. The Cl, Br and I_3_ salt forms all feature short π-geometry contacts between halide atoms and C atoms adjacent to the N atom of the formally positively charged amide group. The C⋯*X* distances are 3.3286 (12), 3.469 (4) and 3.677 (4) Å for the Cl, Br and I_3_ salts, respectively. For Cl and Br, these distances are to the carbonyl C atom, but for I_3_, the closest contact is to atom C1 of the phenyl ring.

**Figure 11:**
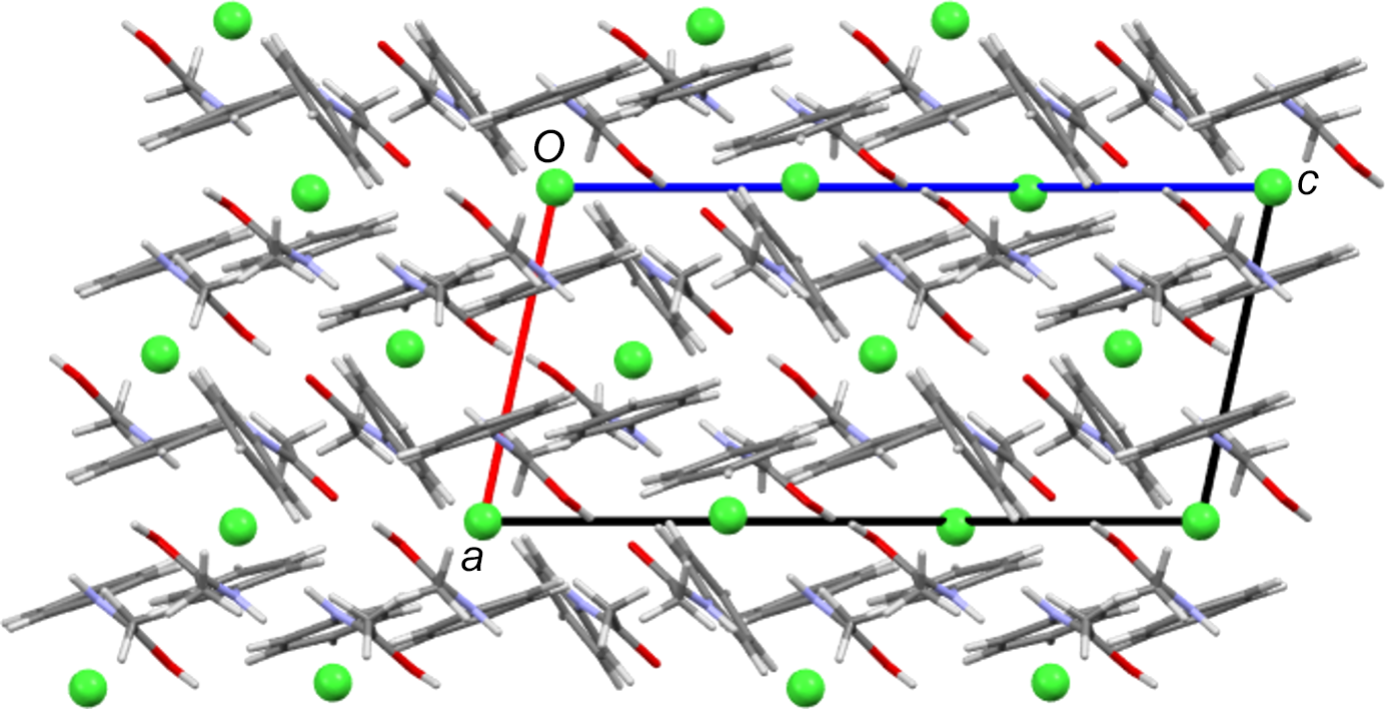
The packing structure of [(ACT)_2_H][Cl], viewed along the *b* axis.

**Figure 13:**
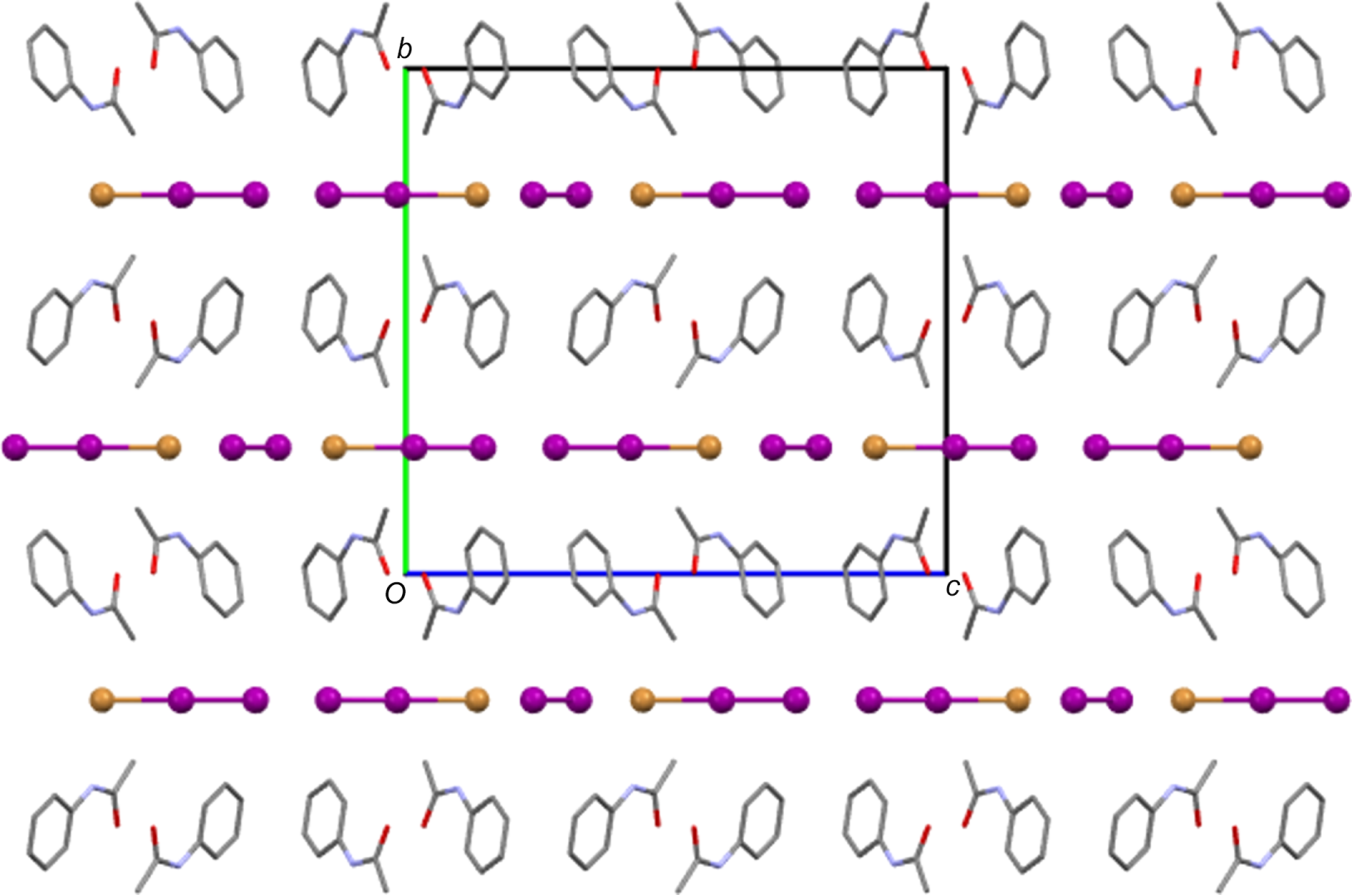
The packing structure of [(ACT)_2_H][I_2_Br]·0.5I_2_, with H atoms and minor disorder com­ponents omitted for clarity. The view is down the *a* axis. Note the layers of halide atoms and ACT fragments that alternate along the *b* direction.

In many ways, the structures of the Cl and Br salt forms are very similar. Both contain three crystallographically independent ACT fragments, two of which have twisted conformations, and two halide sites, one of which is a crystallographic centre of symmetry. As Table 7 ▸ shows, they also feature the same types of inter­molecular inter­actions. Despite this, the two salt forms have different packing structures. Comparing Figs. 11 ▸ and 12 ▸ shows that the Br salt forms a layered structure with alternating layers of anions and bilayers of ACT species, whilst in the Cl salt structure, there are no com­plete anion layers due to their inter­ruption by amide fragments. Of the other structures, the I_3_ and mixed I/Br salt forms give structures with alternate monolayers of anions and organic species (see Fig. 13 ▸), whilst the BF_4_ salt does not form layers.

## Summary

The structures of five anhydrous hemi-protonated salt forms of the simple amide ACT are presented. That no fully protonated forms were isolated is a fundamental difference from ACT’s close congener PAR. All five structures are based around O—H⋯O-contacted ACT(H)–ACT dimers that further link into one-dimensional hy­dro­gen-bonded chains through N—H⋯anion hy­dro­gen bonds. Both amide bond lengths and bond angles change upon protonation. As expected from resonance considerations, the C=O bond lengthens and the C—N bond shortens. The magnitude of these deviations from the geometry of neutral ACT are in line with similar changes seen in hemi-protonated PAR but less than those seen in fully protonated PAR salts. That care should be taken in ascribing all observed changes to protonation is shown by the C—N—C angle. Here large changes seem to be associated more with a change in conformation between planar and twisted units than they are with protonation of the amide.

## Supplementary Material

Crystal structure: contains datablock(s) ACTHCl, ACTHBr, ACTHI3, ACTHBF4, ACTHI2Br, global. DOI: 10.1107/S2053229624007332/ef3058sup1.cif

Structure factors: contains datablock(s) ACTHCl. DOI: 10.1107/S2053229624007332/ef3058ACTHClsup2.hkl

Structure factors: contains datablock(s) ACTHBr. DOI: 10.1107/S2053229624007332/ef3058ACTHBrsup3.hkl

Structure factors: contains datablock(s) ACTHI3. DOI: 10.1107/S2053229624007332/ef3058ACTHI3sup4.hkl

Structure factors: contains datablock(s) ACTHBF4. DOI: 10.1107/S2053229624007332/ef3058ACTHBF4sup5.hkl

Structure factors: contains datablock(s) ACTHI2Br. DOI: 10.1107/S2053229624007332/ef3058ACTHI2Brsup6.hkl

Supporting information file. DOI: 10.1107/S2053229624007332/ef3058ACTHClsup7.cml

Supporting information file. DOI: 10.1107/S2053229624007332/ef3058ACTHBrsup8.cml

Supporting information file. DOI: 10.1107/S2053229624007332/ef3058ACTHI3sup9.cml

Supporting information file. DOI: 10.1107/S2053229624007332/ef3058ACTHBF4sup10.cml

Supporting information file. DOI: 10.1107/S2053229624007332/ef3058ACTHI2Brsup11.cml

CCDC references: 2372822, 2372823, 2372824, 2372825, 2372826

## Figures and Tables

**Figure 2:**
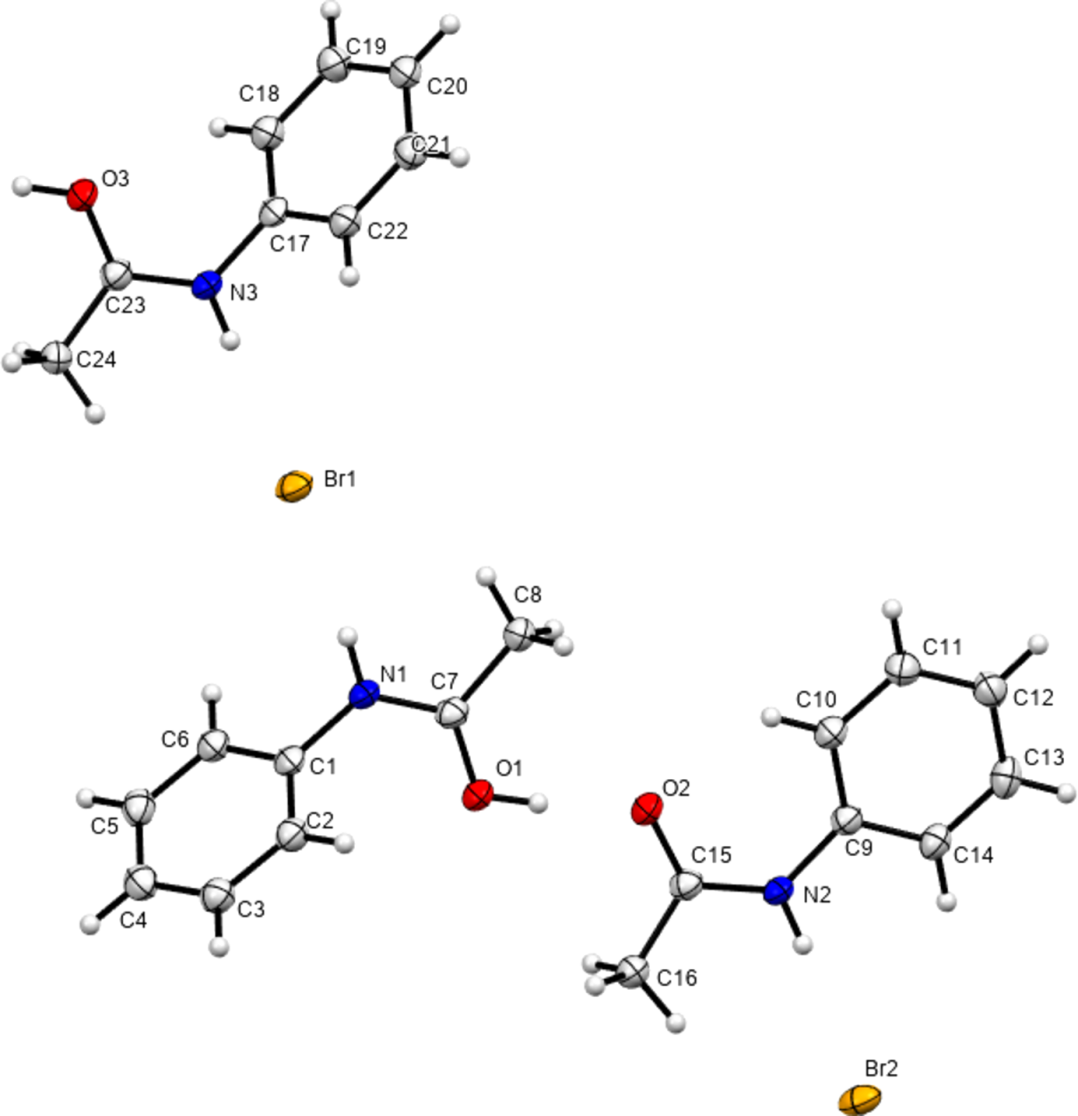
Contents of the asymmetric unit of [(ACT)_2_H][Br].

**Figure 3:**
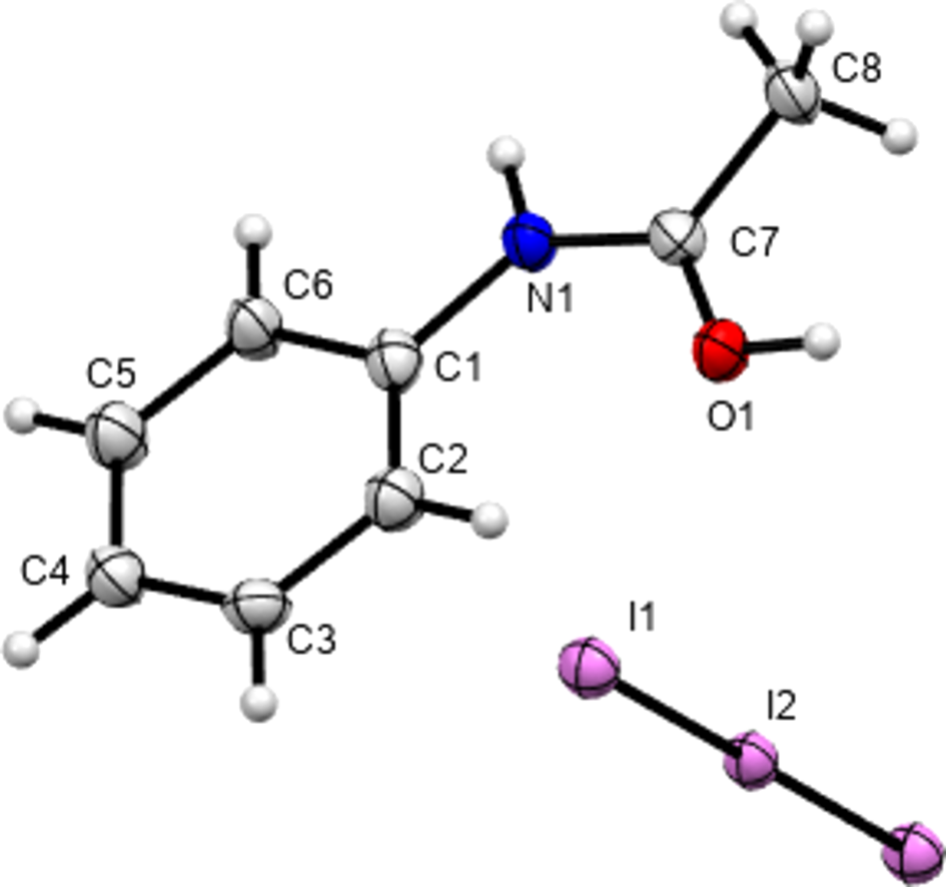
Contents of the asymmetric unit of [(ACT)_2_H][I_3_], expanded to show the com­plete triiodide anion.

**Figure 4:**
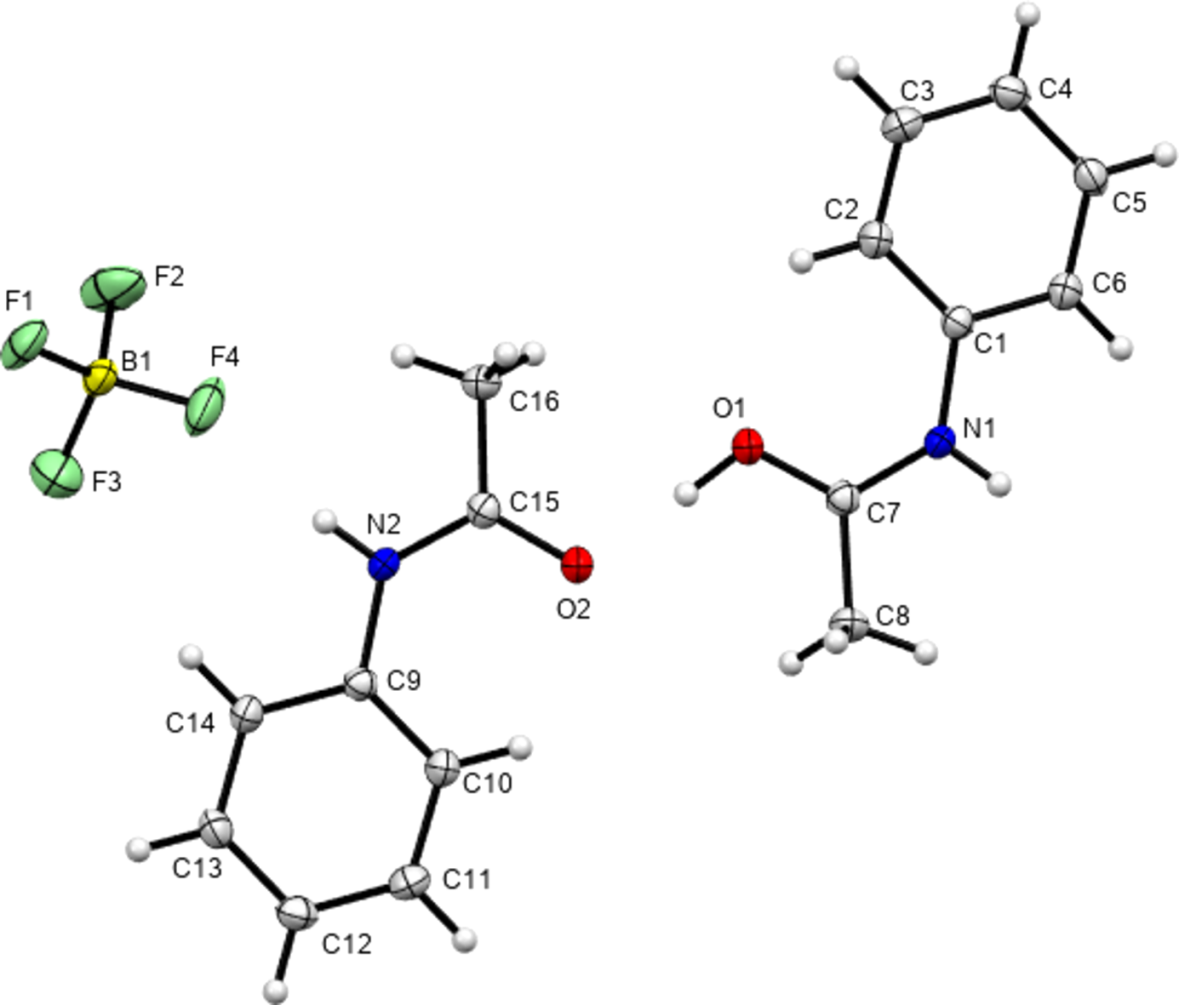
Contents of the asymmetric unit of [(ACT)_2_H][BF_4_].

**Figure 5:**
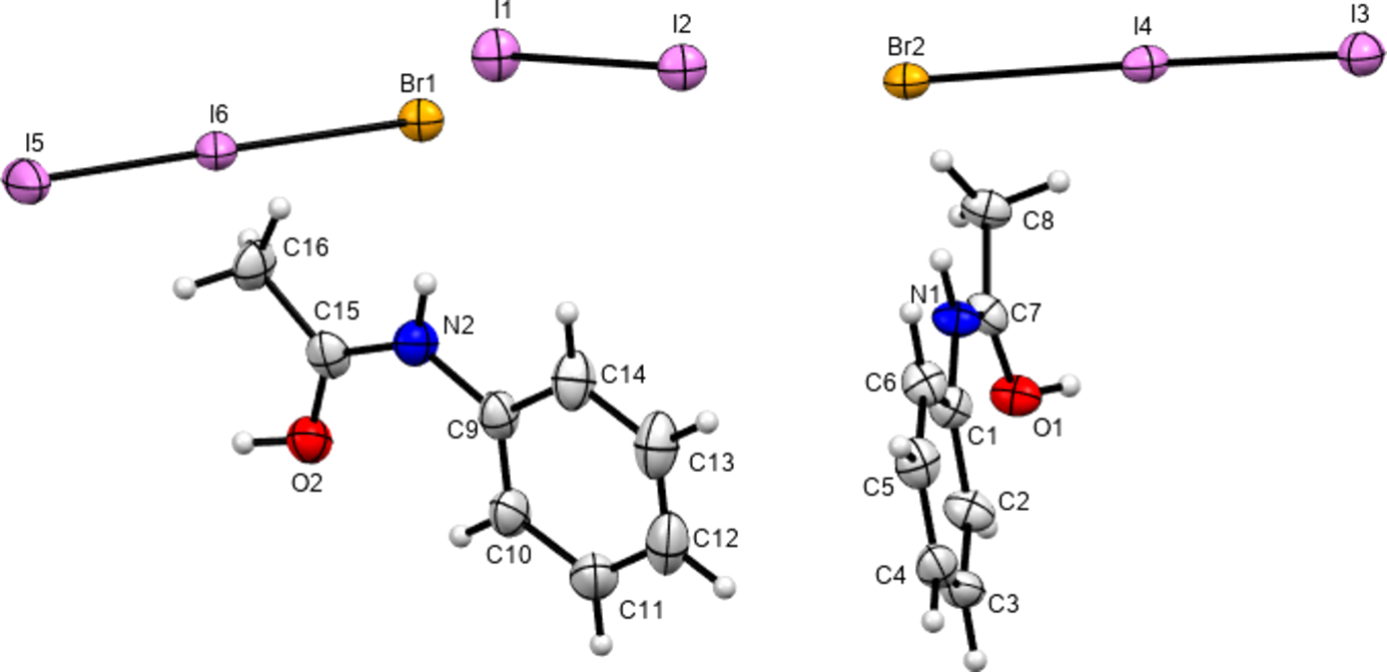
Contents of the asymmetric unit of [(ACT)_2_H][I_2_Br]·0.5I_2_, with atoms of the minor disorder positions omitted for clarity.

**Figure 12:**
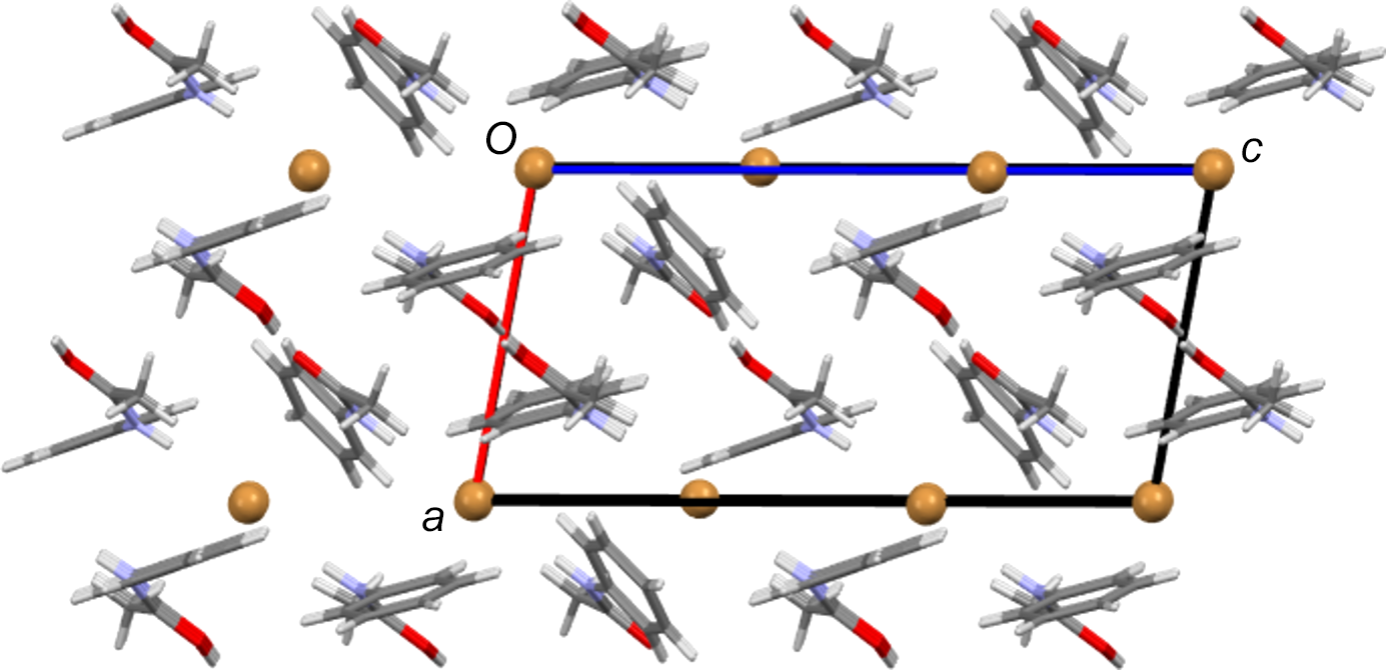
The packing structure of [(ACT)_2_H][Br], viewed along the *b* axis. Note the layers of halide ions and bilayers of ACT fragments that alternate along the *a* direction.

**Table:** Experiments were carried out at 100 K with Cu *K*α radiation using a Rigaku Synergy-i diffractometer. H atoms were treated by a mixture of independent and constrained refinement.

	[(ACT)_2_H][Cl]	[(ACT)_2_H][Br]	[(ACT)_2_H][I_3_]
Crystal data			
Chemical formula	C_8_H_10_NO^+^·Cl^−^·C_8_H_9_NO	C_8_H_10_NO^+^·Br^−^·C_8_H_9_NO	C_8_H_10_NO^+^·I_3_^−^·C_8_H_9_NO
*M*_r_	306.78	351.24	652.03
Crystal system, space group	Monoclinic, *P*2_1_/*n*	Triclinic, *P* 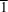	Triclinic, *P* 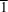
*a*, *b*, *c* (Å)	7.7936 (1), 18.3639 (2), 16.3922 (1)	7.8794 (2), 9.7748 (3), 16.2720 (4)	7.3766 (2), 8.8131 (3), 9.3226 (2)
α, β, γ (°)	90, 102.245 (1), 90	104.385 (3), 98.342 (2), 96.386 (2)	113.612 (2), 103.144 (2), 104.263 (3)
*V* (Å^3^)	2292.69 (4)	1186.86 (6)	500.61 (3)
*Z*	6	3	1
μ (mm^−1^)	2.26	3.59	36.86
Crystal size (mm)	0.20 × 0.15 × 0.12	0.18 × 0.04 × 0.04	0.32 × 0.08 × 0.05

Data collection			
Absorption correction	Multi-scan (*CrysAlis PRO*; Rigaku OD, 2023 ▸)	Analytical [*CrysAlis PRO* (Rigaku OD, 2023 ▸), based on expressions derived by Clark & Reid (1995 ▸)]	Gaussian (*CrysAlis PRO*; Rigaku OD, 2023 ▸)
*T*_min_, *T*_max_	0.725, 1.000	0.668, 0.890	0.034, 0.480
No. of measured, independent and observed [*I* > 2σ(*I*)] reflections	23203, 4444, 4113	8381, 8381, 7175	7238, 1900, 1846
*R*_int_	0.033	Not applicable	0.054
(sin θ/λ)_max_ (Å^−1^)	0.615	0.615	0.615

Refinement			
*R*[*F*^2^ > 2σ(*F*^2^)], *wR*(*F*^2^), *S*	0.031, 0.088, 1.04	0.054, 0.164, 1.11	0.038, 0.107, 1.10
No. of reflections	4444	8381	1900
No. of parameters	309	310	115
No. of restraints	2	5	2
Δρ_max_, Δρ_min_ (e Å^−3^)	0.26, −0.30	0.73, −0.90	1.24, −1.79

**Table:** 

	[(ACT)_2_H][BF_4_]	[(ACT)_2_H][I_2_Br]·0.5I_2_
Crystal data		
Chemical formula	C_8_H_10_NO^+^·BF_4_^−^·C_8_H_9_NO	C_8_H_10_NO^+^·I_2_Br^−^·C_8_H_9_NO·0.5I_2_
*M*_r_	358.14	735.18
Crystal system, space group	Triclinic, *P* 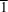	Monoclinic, *P*2_1_/*m*
*a*, *b*, *c* (Å)	7.1070 (1), 9.5381 (1), 13.3592 (1)	5.8956 (1), 18.6224 (4), 19.9632 (4)
α, β, γ (°)	98.765 (1), 98.034 (1), 105.977 (1)	90, 91.970 (2), 90
*V* (Å^3^)	844.56 (2)	2190.47 (7)
*Z*	2	4
μ (mm^−1^)	1.05	36.46
Crystal size (mm)	0.15 × 0.15 × 0.10	0.24 × 0.08 × 0.06

Data collection		
Absorption correction	Multi-scan (*CrysAlis PRO*; Rigaku OD, 2023 ▸)	Analytical [*CrysAlis PRO* (Rigaku OD, 2023 ▸), based on expressions derived by Clark & Reid (1995 ▸)]
*T*_min_, *T*_max_	0.816, 1.000	0.014, 0.164
No. of measured, independent and observed [*I* > 2σ(*I*)] reflections	16151, 3261, 3204	21645, 4338, 3762
*R*_int_	0.026	0.096
(sin θ/λ)_max_ (Å^−1^)	0.615	0.615

Refinement		
*R*[*F*^2^ > 2σ(*F*^2^)], *wR*(*F*^2^), *S*	0.036, 0.099, 1.04	0.052, 0.151, 1.08
No. of reflections	3261	4338
No. of parameters	241	245
No. of restraints	1	2
Δρ_max_, Δρ_min_ (e Å^−3^)	0.50, −0.29	1.50, −1.86

Computer programs: *CrysAlis PRO* (Rigaku OD, 2019 ▸), *SHELXT* (Sheldrick, 2015*a* ▸), *SHELXL2018* (Sheldrick, 2015*b* ▸), *Mercury* (Macrae *et al.*, 2020 ▸) and *SHELXL2018* in *WinGX* (Farrugia, 2012 ▸).

**Table 2:** Hydrogen-bond geometry (Å, °) for [(ACT)_2_H][Cl][Chem scheme1]

*D*—H⋯*A*	*D*—H	H⋯*A*	*D*⋯*A*	*D*—H⋯*A*
N1—H1N⋯Cl2^i^	0.854 (18)	2.237 (19)	3.0899 (11)	176.6 (16)
N2—H2N⋯Cl1	0.851 (18)	2.297 (18)	3.1464 (10)	177.0 (16)
N3—H3N⋯Cl2	0.844 (18)	2.275 (18)	3.1189 (11)	177.6 (16)
O1—H1*H*⋯O2	0.90 (1)	1.57 (1)	2.4650 (12)	175 (3)
O3—H2*H*⋯O3^ii^	0.88 (1)	1.56 (1)	2.4370 (16)	176 (7)

Symmetry codes: (i) 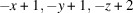; (ii) 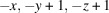.

**Table 3:** Hydrogen-bond geometry (Å, °) for [(ACT)_2_H][Br][Chem scheme1]

*D*—H⋯*A*	*D*—H	H⋯*A*	*D*⋯*A*	*D*—H⋯*A*
N1—H1N⋯Br1	0.88 (1)	2.35 (1)	3.228 (3)	176 (4)
N2—H2N⋯Br2	0.88 (1)	2.44 (1)	3.316 (3)	176 (4)
N3—H3N⋯Br1	0.88 (1)	2.39 (2)	3.256 (3)	174 (4)
O1—H1*H*⋯O2	0.89 (1)	1.57 (2)	2.454 (4)	172 (6)
O3—H2*H*⋯O3^i^	0.88 (1)	1.55 (2)	2.428 (5)	173 (16)

Symmetry code: (i) 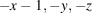.

**Table 4:** Hydrogen-bond geometry (Å, °) for [(ACT)_2_H][I_3_][Chem scheme1]

*D*—H⋯*A*	*D*—H	H⋯*A*	*D*⋯*A*	*D*—H⋯*A*
N1—H1N⋯I1^i^	0.88 (1)	2.82 (1)	3.700 (3)	178 (5)
O1—H1*H*⋯O1^ii^	0.88 (1)	1.55 (2)	2.430 (6)	175 (15)

Symmetry codes: (i) 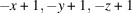; (ii) 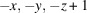.

**Table 5:** Hydrogen-bond geometry (Å, °) for [(ACT)_2_H][BF_4_][Chem scheme1]

*D*—H⋯*A*	*D*—H	H⋯*A*	*D*⋯*A*	*D*—H⋯*A*
N1—H1N⋯F1^i^	0.854 (18)	2.007 (19)	2.8606 (13)	177.6 (16)
N2—H2N⋯F4	0.859 (17)	2.033 (18)	2.8744 (14)	166.0 (15)
O1—H1*H*⋯O2	0.91 (1)	1.53 (1)	2.4333 (12)	174 (3)

Symmetry code: (i) 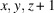.

**Table 6:** Hydrogen-bond geometry (Å, °) for [(ACT)_2_H][I_2_Br]·0.5I_2_[Chem scheme1]

*D*—H⋯*A*	*D*—H	H⋯*A*	*D*⋯*A*	*D*—H⋯*A*
N1—H1N⋯Br2	0.88 (1)	2.49 (1)	3.367 (5)	175 (7)
N2—H2N⋯Br1	0.88 (1)	2.69 (2)	3.546 (10)	164 (7)
O1—H1*H*⋯O1^i^	0.88	1.62	2.442 (10)	155
O2—H2*H*⋯O2^ii^	0.88	1.58	2.434 (10)	164

Symmetry codes: (i) 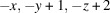; (ii) 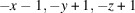.

**Table 7:** Inter­molecular inter­actions found in hemi-protonated ACT structures

Anion	O—H⋯O dimer	Hydrogen-bonded chain	π-anion	π–π	Layering
Cl	Yes	Yes	Yes	No	No
Br	Yes	Yes	Yes	No	Yes
I_3_	Yes	Yes	Yes	Yes	Yes
BF_4_	Yes	Yes	No	Yes	No
I_2_Br	Yes	Yes	No	Yes	Yes
